# Src-Family Protein Kinase Inhibitors Suppress MYB Activity in a p300-Dependent Manner

**DOI:** 10.3390/cells11071162

**Published:** 2022-03-30

**Authors:** Abhiruchi Biyanee, Maria V. Yusenko, Karl-Heinz Klempnauer

**Affiliations:** Institute for Biochemistry, Westfälische-Wilhelms-Universität, D-48149 Münster, Germany; biyanee.abhiruchi@gmail.com (A.B.); m.yusenko@yahoo.com (M.V.Y.)

**Keywords:** MYB inhibitor, acute myeloid leukemia, protein kinase inhibitor

## Abstract

Recent studies have disclosed transcription factor MYB as a potential drug target for malignancies that are dependent on deregulated MYB function, including acute myeloid leukemia (AML) and adenoid cystic carcinoma (ACC). Although transcription factors are often regarded as undruggable, successful targeting of MYB by low-molecular-weight compounds has recently been demonstrated. In an attempt to repurpose known drugs as novel MYB-inhibitory agents, we have screened libraries of approved drugs and drug-like compounds for molecules with MYB-inhibitory potential. Here, we present initial evidence for the MYB-inhibitory activity of the protein kinase inhibitors bosutinib, PD180970 and PD161570, that we identified in a recent screen. We show that these compounds interfere with the activity of the MYB transactivation domain, apparently by disturbing the ability of MYB to cooperate with the coactivator p300. We show that treatment of the AML cell line HL60 with these compounds triggers the up-regulation of the myeloid differentiation marker CD11b and induces cell death. Importantly, we show that these effects are significantly dampened by forced expression of an activated version of MYB, confirming that the ability to suppress MYB function is a relevant activity of these compounds. Overall, our work identifies several protein kinase inhibitors as novel MYB-inhibitory agents and suggests that the inhibition of MYB function may play a role in their pharmacological impact on leukemic cells.

## 1. Introduction

Recent work has highlighted the role of transcription factor MYB in acute myeloid leukemia (AML) and has suggested MYB as a potential drug target for AML and other malignancies associated with deregulated MYB function [1,2,3,4,5]. AML is a common and aggressive type of leukemia that affects children and adults and has a poor prognosis, especially for older patients. In AML, MYB plays a critical role as a master regulator of oncogenic transcriptional programs for the self-renewal and maintenance of leukemic cells [6,7]. MYB has also been implicated in the development of T-cell acute lymphoblastic leukemia (T-ALL), due to duplication or translocation affecting the *MYB* locus. In addition, point mutations that generate MYB-responsive super-enhancers upstream of the *TAL1* or *LMO2* oncogenes have been described in T-ALL patients [8,9,10,11,12]. Finally, recurrent translocations resulting in *MYB-NFIB* gene fusions have been identified as key drivers of adenoid cystic carcinoma (ACC), a slow-growing aggressive cancer often arising in the salivary glands [13]. Recent evidence has suggested that the high expression of MYB fusion proteins in ACC cells is due to an oncogenic feedback loop caused by the re-direction of an MYB-dependent super-enhancer towards the *MYB* gene as a result of the translocation [14].

*MYB* is the cellular progenitor of the v-*myb* oncogene of avian myeloblastosis virus that causes myelomonocytic leukemia in chickens [15]. The cellular *MYB* gene acts as a transcriptional regulator that is highly expressed in hematopoietic progenitor cells and performs crucial functions in the development and homeostasis of the hematopoietic system [16]. Transcriptional activation of target genes by MYB is highly dependent on the cooperation with the co-activator p300. p300 interacts via its so-called KIX-domain with a conserved LXXLL-motif, which is located in the MYB transactivation domain [17]. Mutation of Leu-302 or Met-305 of the LXXLL-motif weaken the binding of p300 and result in decreased MYB activity, demonstrating the role of the LXXLL-motif for MYB activity [18,19,20].

Recently, MYB has attracted attention as a potential drug target for MYB-dependent malignancies [1,3,4,21]. Because AML cells depend on higher levels of MYB activity than normal hematopoietic progenitor cells, they are more vulnerable to MYB inhibition than their normal counterparts [6]. Pioneering studies have found that low-molecular-weight compounds or engineered peptide-drugs can be used to disrupt the MYB/p300 interaction, showing that MYB is a druggable transcription factor [22,23,24,25]. Importantly, these studies have confirmed that blocking MYB activity is effective against AML in an in vivo mouse model. Furthermore, targeting of MYB also inhibited the proliferation of ACC cells in vitro and in mouse models [4,26,27,28]. Thus, pharmacological inhibition of MYB may be a relevant therapeutic strategy against MYB-dependent malignancies.

To expand the repertoire of potential MYB-inhibitors, we have recently screened libraries of approved drugs and related drug-like compounds for MYB-inhibitory agents, using a MYB reporter cell line [29]. Here, we present the initial characterization of three protein kinase inhibitors as candidate MYB-inhibitory compounds.

## 2. Materials and Methods

### 2.1. Cells

Hek293T is non-hematopoietic adherent human cell line grown in DMEM plus 10% FCS. HL60 is human myeloid leukemia cell line cultivated in RPMI1640 medium supplemented with 10% FCS. The cell lines were originally obtained from ATCC and were free of mycoplasma contamination. HL60 cells expressing a C-terminally truncated MYB (MYB-CT3) were generated by lentiviral infection as described before [29]. Control HL60 cells were transfected with an “empty” lentivirus.

### 2.2. Library Screening

The HEK-MYB-Luc and HEK-Luc reporter cell lines have been described before [29]. Library screening of the Selleck FDA-approved Drug Library (SelleckChem, Houston, TX, USA) and the LOPAC-1280 library (Sigma-Aldrich, München, Germany), covering together more than 4000 FDA-approved drugs and compounds with annotated activities, was performed in 384-well plates as described before [28]. In brief, drugs were added at a final concentration of 10 μM to wells containing HEK-MYB-Luc cells pretreated with doxycycline. The luciferase-based reporter signals were detected with the Steady-Glo luciferase kit (Promega, Madison, WI, USA) after 24 h of incubation, using a TECAN microplate reader. Candidate MYB-inhibitory compounds were re-tested in triplicates in HEK-MYB-Luc and HEK-Luc cells at 10 μM concentration. Selected compounds were then subjected to IC_50_ determinations with compound concentrations ranging from 20 μM to 0.01 μM. For further analysis, selected hit compounds were obtained from Sigma-Aldrich and SelleckChem and stored as 10mM stock solutions (in DMSO) at −70 °C.

### 2.3. Expression Vectors

Expression vectors for MYB, MYB-2KR, MYB-CT3, and Gal4-CT3 have been described before [23,30]. M303V and L302A mutant derivatives of MYB-CT3 were obtained by site-directed mutagenesis. The MYB- and Gal4-inducible luciferase reporter plasmids pGL4-5xMRE(GG)-Myc (containing 5 tandem copies of a MYB binding site upstream of a core promoter) and pG5E4-38Luc (containing 5 copies of the Gal4 binding site upstream of the minimal adenovirus E4 promoter) have been described [31,32].

### 2.4. Electrophoretic Mobility Shift Assays (EMSA)

In vitro DNA-binding assays using a radiolabeled double-stranded oligonucleotide with a high affinity MYB binding site were carried out as described before [33]. Nuclear extracts were prepared from non-transfected HEK293T cells and from HEK293T cells transfected with expression vector for MYB-CT3 and incubated for 16 h in the absence or presence of the relevant compounds.

### 2.5. Transfections

Transfection of HEK293T cells by calcium-phosphate co-precipitation and reporter assays were performed as previously described [34].

### 2.6. Flow Cytometry

Approximately 10^6^ HL60 cells were cultured for 2 days in RPMI 1640 medium containing the desired concentration of the kinase inhibitors. Control cells were incubated without compound. The cells were then analyzed by flow cytometry for CD11b expression by using PE/Cy7-labeled anti-human CD11b (ICRF44, BioLegend, Biozol, Eching, Germany), using a FC 500 Cytometer (Beckman Coulter, Krefeld, Germany). Dead cells were determined by staining them without fixation with propidium iodide (PI). CXP software (Beckman Coulter, Krefeld, Germany) was used for subsequent analysis.

### 2.7. Western Blot Analysis

Protein samples were analyzed by SDS-PAGE and Western blotting with the following antibodies: anti-Myb (5E11, [35]), anti-β-actin (Sigma-Aldrich, München, Germany; AC-15), and anti-Gal4 (SantaCruz Biotechnology, Heidelberg, Germany; RK5C1).

### 2.8. Statistical Analysis

All experiments subjected to statistical analysis were performed at least three times with independent replicates in each experiment. Data were shown as mean ± standard deviation, which reflects the variation within each group. Statistical differences between groups were calculated by the two-tailed Student’s *t*-test. Values of *p* < 0.05 were considered as statistically significant.

## 3. Results

### 3.1. Identification of Protein Kinase Inhibitors Bosutinib, PD161570, and PD180970 as MYB-Inhibitory Agents

**Figure 1:**
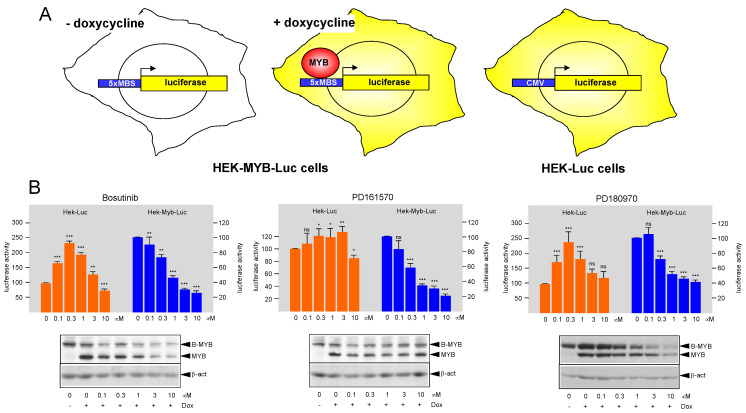
MYB inhibitory activity of selected protein kinase inhibitors. (**A**) Schematic illustration HEK-MYB-Luc and HEK-Luc cells. HEK-MYB-Luc cells carry a stably transfected artificial MYB-inducible luciferase reporter gene and a doxycycline-inducible expression system for human MYB-2KR. HEK-Luc cells carry a constitutively active luciferase expression vector. (**B**) HEK-Luc and HEK-MYB-Luc cells were treated for 16 h with Bosutinib, PD161570, and PD180970 at the indicated concentrations. Bars show the average luciferase activity of the cells relative to cells treated only with doxycycline. The bottom panels show the expression of MYB, B-MYB (cross-reacting with the anti-MYB antibody) and β-actin. Asterisks indicate statistical significance (ns: not significant; * *p* < 0.05; ** *p* < 0.01; *** *p* < 0.001; Student’s *t*-test).

We have recently employed the luciferase reporter cell-lines HEK-MYB-Luc and HEK-Luc to screen libraries of approved drugs and drug-like compounds for molecules that inhibit MYB activity [28]. As illustrated schematically in Figure 1A, HEK-MYB-Luc cells carry a stably integrated luciferase reporter gene that contains several high-affinity MYB binding sites fused upstream of a minimal promoter. The cells also harbor a doxycycline-inducible expression system for an activated version of human MYB, referred to as MYB-2KR [29]. HEK-Luc cells harbor a luciferase reporter gene driven by the CMV promoter to serve as control for unspecific effects on luciferase expression. Compound screening was performed as described before [28], employing 10 μM compound concentrations and using 384-well microtiter plates. The previously identified MYB-inhibitory compound monensin [27] served as positive control to assess the reproducibility of the assay. Inhibitory compounds were confirmed by a secondary screen and were subjected to IC_50_ analysis with compound concentrations ranging from 0.01 μM to 20 μM in duplicate measurements. Overall, we identified several proteasome inhibitors, HDAC inhibitors, and protein kinase inhibitors as potential MYB-inhibitory agents. Recently, characterized the ability of proteasome and HDAC inhibitors to suppress MYB activity in detail [28,36]. Here, we have focused on the protein kinase inhibitors that showed MYB-inhibitory activity. We identified four compounds classified as SRC/BCR-ABL inhibitors (Bosutinib, Dasatinib, Nilotinib, and PD180970), as well as the FGFR1/SRC inhibitor PD161570. All of them showed EC50 concentrations for MYB inhibition around 1 μM or slightly higher. We selected three compounds for further studies, namely, Bosutinib and PD180970, as examples of SRC/BCR-ABL class of kinase inhibitors and PD161570 as a FGFR1/SRC inhibitor. Figure 1B shows representative luciferase assays of HEK-MYB-Luc and HEK-Luc cells treated with different concentrations of the selected compounds, confirming that they inhibit MYB activity at low micromolar concentrations. Luciferase activity in the HEK-Luc cells was only affected slightly by PD161570, whereas in case of Bosutinib and PD180970, it first increased at low compound concentrations and then decreased again at concentrations of 1 μM or higher. While the reason for the increase at low compound concentration was not studied further, the decrease at higher concentrations might be due to non-specific toxic effects on the cells. In any case, the observation that luciferase activity in HEK-MYB-Luc cells already decreased at the low compound concentrations in all three cases suggested that they all exert MYB-specific inhibitory effects. We also analyzed total cell extracts of HEK-MYB-Luc cells by Western blotting for MYB and β-actin expression (see bottom panels of Figure 1B). We used an antibody against the MYB DNA-binding domain that cross-reacts with B-MYB, explaining why both MYB and B-MYB are displayed in the Western blots. Consistent with the design of the HEK-MYB-Luc cells, MYB was not expressed in the absence of doxycycline.

**Figure 2:**
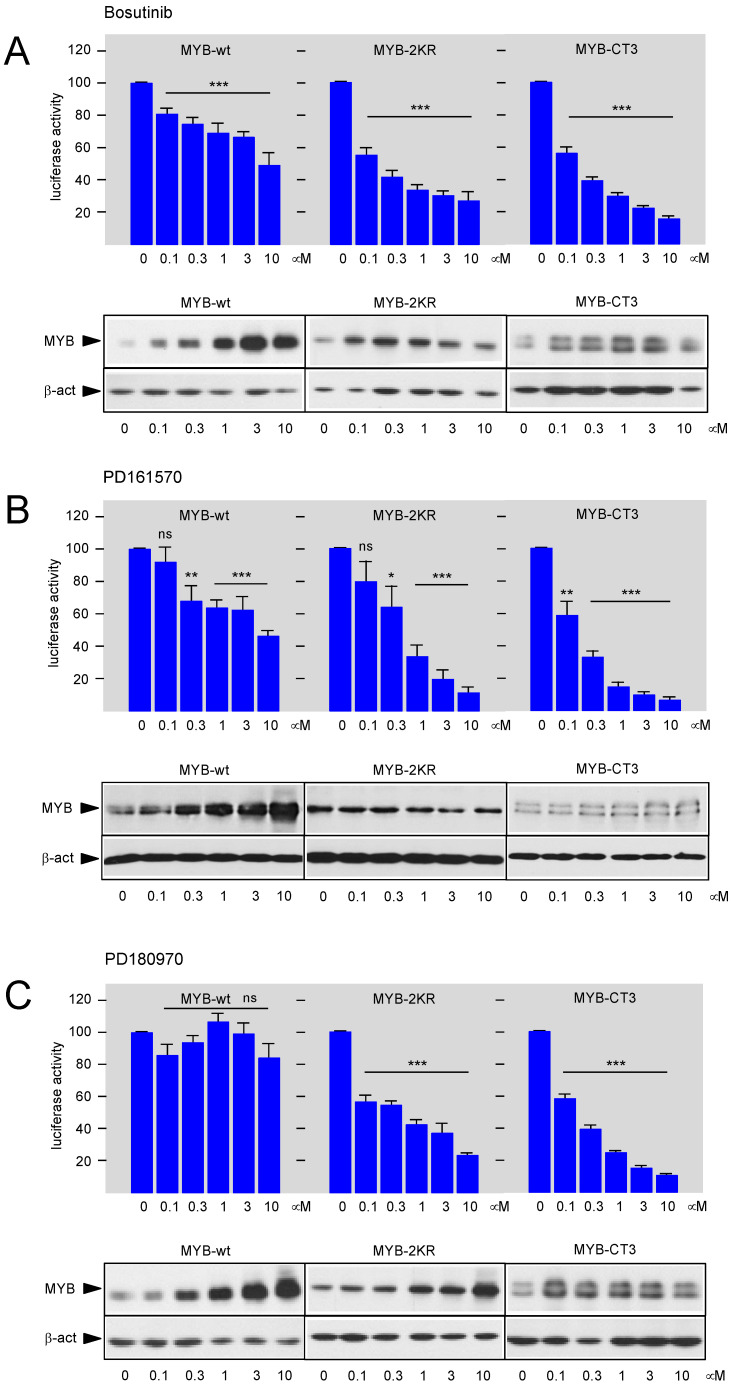
Comparison of the inhibitory activity of selected compounds towards wild-type MYB, MYB-2KR, and C-terminally truncated MYB. HEK293T cells were transfected with expression vectors for wt MYB, MYB-2KR, and MYB-CT3 and the MYB-responsive reporter gene pGL4-5xMRE(GG)-Myc. Transfected cells were distributed in identical aliquots into microtiter plates and treated with the indicated concentrations of Bosutinib (**A**), PD161570 (**B**) or PD180970 (**C**). Cells were harvested after 16 h and used for luciferase assays. Identically treated cells were also analyzed by western blotting to determine MYB expression. Bars depict the average luciferase activity normalized to untreated cells. Asterisks indicate statistical significance (ns: not significant; * *p* < 0.05; ** *p* < 0.01; *** *p* < 0.001; Student’s *t*-test). The bottom shows relevant parts of the protein gels containing MYB, MYB-2KR, MYB-CT3, and β-actin.

Because HEK-MYB-Luc cells express the activated MYB-2KR mutant, we next investigated the effect of the compounds on wild-type MYB. Therefore, we transfected expression vectors for MYB-2KR or wild-type MYB together with the MYB-dependent luciferase reporter transiently into HEK293T cells and assessed the effect of different concentrations of the compounds (Figure 2). As expected, the activity of MYB-2KR was inhibited by all compounds. Wild-type MYB was inhibited less strongly by Bosutinib and PD161570, whereas PD180970 showed no significant inhibition. We noted that wild-type MYB expression was strongly increased at the higher compound concentrations in all cases, which might explain the decreased inhibitory potential of the compounds relative to MYB-2KR. In additional experiments, we also examined the ability of the compounds to inhibit the activity of the C-terminally truncated mutant MYB-CT3. The C-terminal MYB sequences are thought to link MYB to different signaling pathways and thereby to have regulatory functions. For example, the C-terminal domain of MYB is phosphorylated by mitogen-activated protein kinases [37,38,39,40]. We therefore wondered whether signaling pathways that target MYB via its C-terminal domain are affected by the protein kinase inhibitors. However, analysis of MYB-CT3 showed that its activity was strongly inhibited by all compounds tested, thereby excluding that they target MYB via the C-terminal domain (Figure 2). We also noted that the amount of MYB-CT3 expression was not strongly increased when the compound concentration was raised. 

### 3.2. Bosutinib, PD161570, and PD180970 Affect the Function of the MYB Transactivation Domain

**Figure 3:**
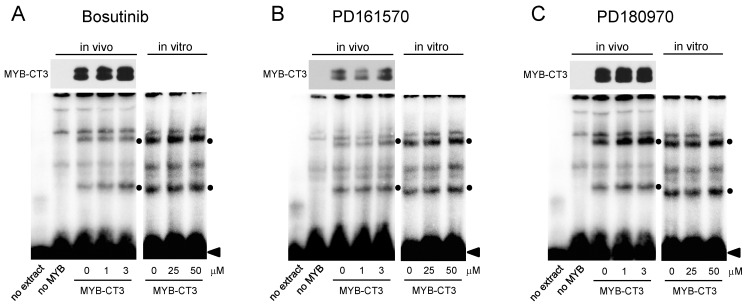
MYB-inhibitory compounds do not inhibit MYB DNA-binding activity. The left parts of each panel show EMSA experiments with nuclear extracts from HEK293T cells transfected without or with expression vector for MYB-CT3 and incubated for 16 h with Bosutinib (**A**), PD161570 (**B**) or PD180970 (**C**) at the indicated concentrations. Nuclear extract from un-transfected HEK293T cells was used as control. Binding assays were performed with a radiolabeled oligonucleotide containing a consensus MYB binding site. A western blot showing MYB-CT3 expression in aliquots of the nuclear extracts is presented at the top. The right part of each panel shows EMSA experiments with nuclear extracts from MYB-CT3-expressing cells supplemented with the indicated compound concentrations during the in vitro binding reactions. Black dots mark MYB-specific protein-DNA-complexes.

Since our data had excluded the possibility that the kinase inhibitors suppress MYB activity via its C-terminal regulatory domain, we focused on the transactivation and DNA-binding domains of MYB as potential targets of the compounds. Nuclear extracts were prepared from MYB-CT3-expressing cells after treating them for 16 h with the relevant compound concentrations. We then subjected the extracts to electrophoretic mobility shift assays (EMSA) to investigate MYB DNA-binding activity. Upon incubating a radiolabeled MYB-binding site oligonucleotide with nuclear extract, we observed several complexes with retarded electrophoretic mobility, two of which (labeled by black dots in Figure 3) were not present when control extracts from un-transfected cells were used. This indicated that these are specific complexes containing MYB-CT3 bound to the MYB-binding site oligonucleotide. We assume that the slower and faster migrating of the MYB-specific complexes are derived from full-length and partially degraded MYB-CT3. Importantly, the EMSA experiments showed no decrease of the MYB-specific complexes when compound concentrations were used that strongly suppress the activity of MYB-CT3 (Figure 3). This indicated that the inhibitory compounds do not disrupt the DNA-binding activity of MYB. We also performed additional EMSA experiments in which the MYB-CT3 containing nuclear extract was supplemented with significantly higher compound concentrations in vitro (see panels marked “in vitro” in Figure 3). However, none of the compounds showed an effect on MYB DNA-binding activity.

**Figure 4:**
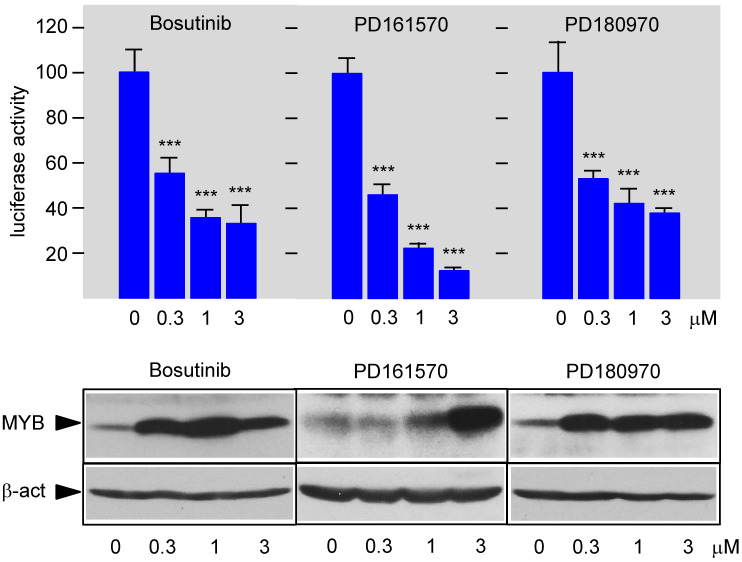
Bosutinib, PD161570, and PD180970 suppress the activity of the MYB transactivation domain. HEK293T cells transfected with an expression vector for a Gal4-CT3 fusion protein and the Gal4-responsive reporter gene pG5E4-38Luc were distributed in microtiter plates and treated for 16 h with the indicated compound concentrations. Luciferase activities are displayed as in Figure 2. The expression of Gal4-CT3 and β-actin is shown in the bottom panels. Asterisks indicate statistical significance (*** *p* < 0.001; Student’s *t*-test).

Since the EMSA experiments excluded that the compounds act by interfering with MYB DNA-binding, we next investigated their impact on the MYB transactivation domain, which is encoded by amino acid sequences located C-terminally to the DNA-binding domain. We replaced the DNA-binding domain of MYB-CT3 by the yeast GAL4 DNA-binding domain and investigated whether the activity of the fusion protein was still inhibited by the compounds. As shown in Figure 4, in each case the activity of the GAL4-MYB fusion protein containing the MYB transactivation domain was inhibited in a concentration-dependent manner. Overall, these experiments demonstrated that all three compounds inhibit MYB activity via its transactivation domain.

### 3.3. Bosutinib, PD161570, and PD180970 Disturb the Stimulation of MYB Activity by the Co-Activator p300

**Figure 5:**
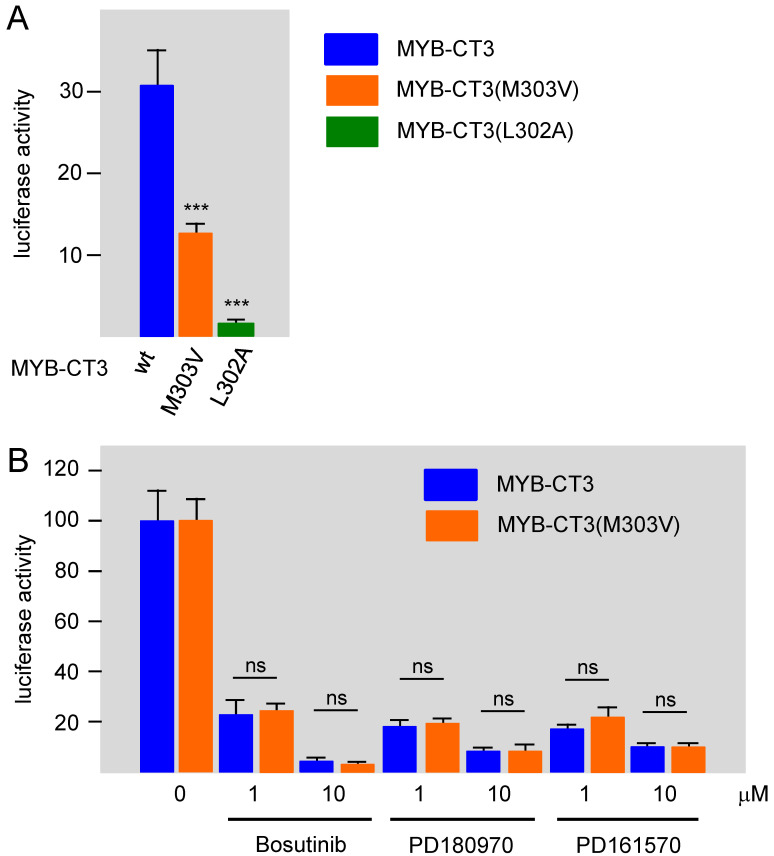
Bosutinib, PD161570, and PD180970 disturb the cooperation between MYB and co-activator p300. (**A**) Luciferase reporter assays of HEK293T cells transfected with the MYB-responsive reporter gene pGL4-5xMRE(GG)-Myc and expression vectors for MYB-CT3, MYB-CT3(M303V), or MYB-CT3(L302A). Asterisks indicate statistical significance (*** *p* < 0.001; Student’s *t*-test). (**B**) HEK293T cells were transfected with reporter gene pGL4-5xMRE(GG)-Myc and expression vectors for MYB-CT3 or MYB-CT3(M303V) and treated without or with the indicated concentrations of the kinase inhibitors for 16 h before harvesting and measuring luciferase activities. To compensate for the difference in activity between MYB-CT3 and the M303V mutant, luciferase activities of untreated cells were normalized to 100 percent. “ns” refers to “not statistically significant” (Student’s *t*-test).

Since the compounds investigated are known inhibitors of protein kinase signaling pathways, we considered the possibility that the activity of the MYB transactivation domain is subject to phosphorylation that regulate its activity. However, to our knowledge, such phosphorylation has not been described, suggesting that the inhibitors do not act on MYB directly but rather exert their effects indirectly via proteins that interact with the transactivation domain. Previous work had shown that the transactivation potential of MYB strongly depends on the direct binding of the co-activator p300 to the transactivation domain [17]. Single amino acid replacements within the MYB transactivation domain, such as methionine-303 by valine (M303V) or leucine-302 by alanine (L302A), are known to weaken the binding of p300 to MYB and to decrease the transactivation potential of MYB [19,41]. As expected, when we introduced these mutations into MYB-CT3, MYB activity was strongly reduced or almost completely abolished (Figure 5A). The almost complete loss of the transactivation potential in case of the L302A mutant, which is in line with the strong disruption of the MYB-p300 interaction [41], suggests that MYB activity under the assay conditions that we are using is completely dependent on p300 or the highly related CREB-binding protein (CBP). This, in turn, means that the inhibitory compounds most likely act by interfering with the cooperation between MYB and p300 (or CBP). To substantiate this conjecture, we employed the M303V mutation to confirm that the inhibitory compounds act in a p300-dependent manner. We reasoned that the relative contribution of a hypothetical p300-independent mechanism to MYB activity would be increased in case of the M303V mutant relative to wild-type MYB because the weaker binding of p300 reduces its contribution to the transactivation potential of the M303V MYB mutant. Thus, a compound that inhibits MYB activity independently of p300 would be expected to have a stronger inhibitory effect on the M303V mutant than on wild-type MYB. Therefore, we compared the inhibitory effects of the kinase inhibitors on wild-type MYB-CT3 and its M303V derivative. To facilitate this comparison, the activities of both versions of MYB-CT3 were normalized to 100 percent and compared side-by-side in Figure 5B. As can be seen, the inhibition at each concentration of the compounds was identical for wild-type and the mutant MYB, supporting the notion that the compounds decrease the stimulation of MYB activity in a p300-dependent manner. 

### 3.4. Bosutinib, PD161570, and PD180970 Induce Expression of Myeloid Differentiation Marker CD11b and Cell Death in HL60 Cells

**Figure 6:**
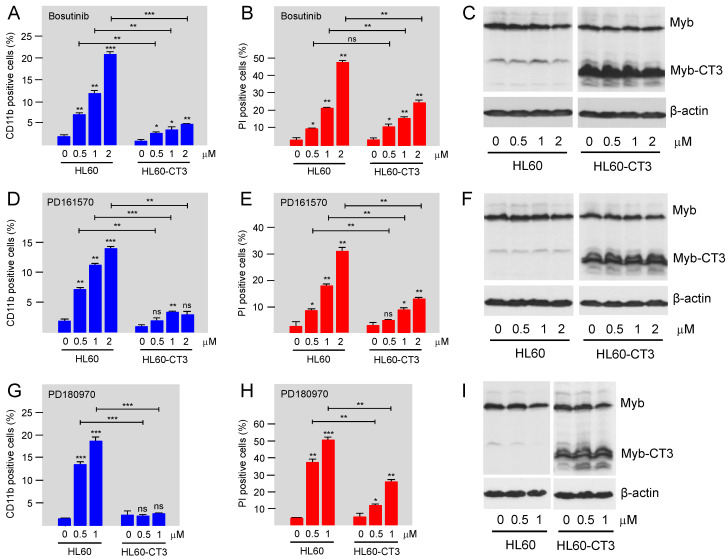
Kinase inhibitors affect HL60 cells in a MYB-dependent manner. HL60 cells infected with a control lentivirus or a lentivirus encoding C-terminally truncated MYB (HL60-CT3) were treated for 48 h with the indicated concentrations of Bosutinib (panels **A** to **C**), PD161570 (panels **D** to **F**), and PD180970 (panels **G** to **I**). The cells were then analyzed by flow cytometry for CD11b expression (panels **A**,**D**,**G**), by staining with propidium iodide (panels **B**,**E**,**H**), and by Western blotting for MYB and β-actin expression (panels **C**,**F**,**I**). Asterisks indicate statistical significance (ns: not significant; * *p* < 0.05; ** *p* < 0.01; *** *p* < 0.001; Student’s *t*-test).

To characterize the MYB-inhibitory potential of the kinase inhibitors in a relevant biologically setting, we studied their effects on the human myeloid leukemia cell line HL60. In myeloid leukemia, MYB is required to maintain the viability of the leukemia cells and to prevent their differentiation. Inhibition of MYB is therefore expected to trigger differentiation and cell death of these cells. Incubation of HL60 cells in the presence of different concentrations of Bosutinib, PD161570, or PD180970 showed that the myeloid differentiation marker CD11b was up-regulated by each compound in a concentration-dependent manner, consistent with the MYB-inhibitory activity of the compounds. In addition, we observed a concentration-dependent loss of cell viability, as measured by staining the live cells with propidium iodide (Figure 6). Propidium iodide intercalates into the cellular DNA and brightly stains the cells only if the integrity of the cellular membrane is disrupted, i.e., when the cells are no longer viable. Since we treated the cells only for 48 h with the compounds, it is open to question whether terminal differentiation would be induced upon longer incubation times. However, it is possible that the simultaneously induced massive cell death eventually interferes with terminal differentiation. To further demonstrate that these effects of the compounds were indeed due to their MYB-inhibitory potential, we performed parallel experiments with HL60 cells ectopically expressing the C-terminally truncated, and thereby activated, MYB-CT3 [42]. Thus, if induction of CD11b expression or cell death were simply caused by inhibition of kinase signaling independently of MYB, we would expect to observe identical changes also in the MYB-CT3-expressing cells. As seen in Figure 6, in the presence of the activated MYB, the induction of CD11b expression as well as of cell death were strongly diminished, indicating that the ability of the compounds that cause these effects was due to a large extent to the inhibition of MYB activity. While this was particularly evident in case of CD11b expression, the induction of cell death was less strongly diminished, suggesting the MYB-independent activities of the compounds were also involved or that higher levels of MYB activity are required to counteract the loss of cell viability by the compound treatment.

## 4. Discussion

Recent investigations into the role of MYB in human leukemia have pin-pointed MYB as a potential drug target for AML and have stimulated research in the use of low-molecular-weight compounds as potential MYB inhibitors. Although transcription factors are generally considered as difficult to target by pharmacological inhibitors, recent proof-of-principle studies by our group and others have demonstrated that MYB is a druggable protein and obtained initial evidence that inhibition of MYB may have relevant therapeutic potential [4,22,23,24,25]. Moreover, possibilities to inhibit MYB by indirect strategies have come into focus [43,44]. This has set the stage for studies aiming to address if already existing drugs can be repurposed as MYB-inhibitory agents and thereby to expand the repertoire of such compounds. With this in mind, we have screened libraries of approved drugs and drug-like compounds for potential MYB inhibitors, using a recently established MYB reporter cell line [29]. This has allowed us to identify several compounds that inhibit MYB activity at low micromolar or nanomolar concentration, including polyether ionophores [27]; proteasome inhibitors [28]; HDAC inhibitors [36]; and, as illustrated by this report, certain inhibitors of tyrosine-specific protein kinases. 

Here, we have presented the initial characterization of the MYB-inhibitory activities of the protein kinase inhibitors Bosutinib, PD180970, and PD161570. Bosutinib and PD180970, as well as Dasatinib and Nilotinib, which also showed MYB-inhibitory activity in our primary screen but have not yet been studied in more detail, are inhibitors of the SRC and BCR-ABL protein kinases. PD161570 is a FGFR1 kinase inhibitor that has also similar potency against SRC kinase [45]. Our initial studies have shown that these compounds inhibit the activity of the MYB transactivation domain and excluded that they affect the DNA-binding activity of MYB or signaling pathways targeting the C-terminal “regulatory” domain of MYB. We have also shown that treatment of HL60 cells with these kinase inhibitors causes up-regulation of the differentiation marker CD11b as well as cell death, both of which are known consequences of decreased MYB activity in AML cells. Induction of differentiation in HL60 cells by Bosutinib and Dasatinib has also been observed previously [46,47] but has not been linked to inhibition of MYB activity before. Importantly, we have shown that forced expression of an activated version of MYB in HL60 cells significantly dampened the response of the cells to the treatment with the protein kinase inhibitors. Overall, this confirms that the inhibition of MYB function as a relevant activity of these compounds in myeloid cells. 

SRC-family kinases are profoundly involved in various signaling pathways whose hyperactivity is frequently observed in leukemia cells and contributes to the development of acute and chronic myeloid leukemia. Because of the co-expression of different SRC kinase family members in leukemia cells, their functional redundancies and the promiscuity of many of the available SRC-family kinase inhibitors makes it a daunting task to unravel the underlying molecular mechanisms we can currently only speculate on how they inhibit MYB activity. Our data indicate that the kinase inhibitors studied here target MYB via its transactivation domain, suggesting that they may primarily affect the function of the MYB co-activator p300/CBP as the main driver of MYB transcriptional activity. Because of the multitude of known p300- or CBP-interacting proteins, p300 and its paralog CBP function as nodal points in the transcriptional network, thus presenting multiple possibilities for how the transcriptional output can be integrated by signaling pathways affecting these co-activators, either directly or indirectly, via interaction partners [48,49,50]. For example, the activity of AIB/SRC-1, a co-activator interacting with the C-terminal domain of p300, is modulated by tyrosine phosphorylation mediated by ABL kinase [51]. Likewise, MAPK-mediated phosphorylation of the C-terminal part of p300 provides further possibilities for how upstream-acting tyrosine-specific kinase inhibitors may affect p300 [52,53,54,55,56]. Recent work has also revealed a nuclear SRC-kinase/p300 signaling axis [57] and shown that inhibition of SRC or ABL Kinases can indirectly impair the HAT activity of p300 [58,59]. Finally, previous work has also suggested that nuclear FGFR1 and p300 may be involved in an integrative signaling module [60].

In summary, these examples illustrate the potential of specific protein kinase inhibitors to modulate the activity of p300 and p300-dependent transcription factors. With respect to MYB, it will be very interesting to decipher these mechanisms in more detail in future work. Furthermore, it will also be very interesting to examine if combinations of MYB-inhibitory protein kinase inhibitors and other MYB-inhibitory agents exert synergistic effects MYB-dependent tumor cells.

## Data Availability

All data are contained in this manuscript.
